# Genetic variants in humanin nuclear isoform gene regions show no association with coronary artery disease

**DOI:** 10.1186/s13104-019-4807-x

**Published:** 2019-11-21

**Authors:** Mall Eltermaa, Maili Jakobson, Meeme Utt, Sulev Kõks, Reedik Mägi, Joel Starkopf

**Affiliations:** 1 Institute of Biomedicine and Translational Medicine, University of Tartu, Tartu, Estonia; 2 Institute of Pharmacy, University of Tartu, Tartu, Estonia; 3 Murdoch University, Murdoch, WA Australia; 4 Perron Institute for Neurological and Translational Science, Nedlands, WA Australia; 5 Institute of Genomics, University of Tartu, Tartu, Estonia; 6 Department of Anaesthesiology and Intensive Care, Tartu University Hospital, Tartu, Estonia

**Keywords:** Humanin, Humanin-like, Coronary artery disease, Association study, Peptide

## Abstract

**Objective:**

Coronary artery disease contributes to noncommunicable disease deaths worldwide. In order to make preventive methods more accurate, we need to know more about the development and progress of this pathology, including the genetic aspects. Humanin is a small peptide known for its cytoprotective and anti-apoptotic properties. Our study looked for genomic associations between humanin-like nuclear isoform genes and coronary artery disease using CARDIoGRAMplusC4D Consortium data.

**Results:**

Lookup from meta-analysis datasets gave single nucleotide polymorphisms in all 13 humanin-like nuclear isoform genes with the lowest *P* value for rs6151662 from the *MTRNR2L2* gene including the 50 kb flanking region in both directions (P-value = 0.0037). Within the gene region alone the top variant was rs78083998 from the *MTRNR2L13* region (meta-analysis P-value = 0.042). None of the found associations were statistically significant after correction for multiple testing. Lookup for expression trait loci in these gene regions gave no statistically significant variants.

## Introduction

Cardiovascular diseases account for most noncommunicable disease (NCD) deaths worldwide [1]. The WHO global action plan [1] for the years 2013–2020 aims to support and promote research in NCD to reduce the global burden via better prevention. Therefore, a lot of effort has been made to better understand the interplay of environmental, behavioural and genetic risk factors in order to make preventive methods more efficient.

Coronary artery disease (CAD) or ischaemic heart disease involves the reduction of blood flow to the heart muscle because of pathological process of atherosclerosis in coronary vessels. The typical presentations are angina pectoris, unstable angina, myocardial infarction and sudden cardiac death [2]. The genetic predisposition to CAD has received increasing attention. For example, in a recent study, 64 novel genetic risk loci for CAD were identified, expanding the total to 161 [3]. There are genes known to have a large effect on, for example, lipoprotein metabolism (e.g. *LDLR*), resulting in increased risk of early-onset coronary disease [4]. However, CAD seems to be an omnigenic disease where all gene-regulatory networks may be interrelated and different tissues may contribute to disease progression [3].

In 2001, a novel gene cDNA, 99% identical to the mitochondrially encoded 16S rRNA gene (*MTRNR2*), was reported to encode a short peptide-humanin [5, 6]. Functionally, it was shown that this peptide binds with insulin-like growth factor-binding protein-3 (IGFBP-3) and Bax protein [7, 8]. Additional studies have revealed that humanin has a cytoprotective function and a role in apoptosis regulation [5–8]. Regarding cardiovascular diseases, this peptide has been shown to be expressed in the endothelial layer of human blood vessels including the mammary artery, atherosclerotic coronary artery, and greater saphenous vein [9]. A lower circulating humanin level has been described in patients with coronary endothelial dysfunction compared to healthy controls [10].

Data from Bodzioch et al. [11] suggested the existence of 13 nuclear-encoded humanin isoforms: humanin-like open reading frames named and numbered *MTRNR2L1* to *MTRNR2L13* after the original humanin *MTRNR2* gene in the mitochondrial genome. This research also showed considerable gene homology between different species. The study showed that *MTRNR2L1*–*MTRNR2L10* were expressed variably in most human tissues, whereas *MTRNR2* expression was considerably higher compared to the isoforms in all the studied tissues. *MTRNR2, MTRNR2L1, MTRNR2L8* and *MTRNR2L9* were highly expressed in the heart muscle suggesting their possible role in this tissue as cytoprotective peptides.

Most research has focused on humanin and its function. Studies done on several subjects have revealed differences in expression of humanin-like isoform genes and their regulation through hypoxia, miRNA or circRNA, to name some of these [12–19]. DNA copy number change in isoform genes has been found in arteries of patients with CHD and metabolic comorbidities [20]. A genome-wide association study on Huntington`s disease and copy number variation studies in classic heterotaxy and schizophrenia have identified variants in humanin-like isoform genes [21–23]. Humanin isoforms have also been studied as potential biomarkers for Alzheimer`s disease-like dementia and Hirschsprung’s disease, suggesting their wider role in physiological processes [24, 25]. Research on cancer subtypes, Dicer-binding genes, genomic mosaicism, extracellular RNA profiles, chromosomal rearrangements and huntingtin interactions has provided insight into networks in which humanin-like isoforms might be of importance [26–31].

Previously, we have shown changes in some humanin-like isoform expression levels in the context of cardiovascular disease [32]. This study design involved adult patients undergoing general anaesthesia before coronary artery bypass grafting; patients were randomly assigned to be ventilated with different FiO_2_ 60 min before operation. Exposure to > 96% oxygen upregulated two of the humanin-like peptide genes—*MTRNR2L2* and *MTRNR2L8*—in the right atrial appendage tissue sample. Whether this change was the consequence of the different oxygen levels or whether there could be possible single nucleotide variations (SNVs) in isoform genes responsible for the observed effect after oxygen exposure, is not clear. Therefore, we decided to look for possible single nucleotide polymorphisms (SNPs) in gene regions of humanin-like peptides.

To study the possible association between coronary artery disease and genetic variation in humanin-like nuclear isoform peptide coding genes, we performed a lookup within all 13 humanin-like-peptide-coding genomic regions using publicly accessible meta-analysis data for coronary artery disease from the CARDIoGRAMplusC4D Consortium (Coronary ARtery DIsease Genome wide Replication and Meta-analysis (CARDIoGRAM) plus The Coronary Artery Disease (C4D) Consortium) [33]. We also analysed tissue-specific expression influences at the DNA level using data from the GTEx expression quantitative trait loci (eQTL) database [34].

## Main text

### Methods

The aim of this study was to conduct a lookup from publicly available meta-analysis data on genetic associations between single nucleotide variants in 13 humanin-like nuclear isoform genes and coronary artery disease.

#### Materials

Genome-wide SNP association lookup was conducted using CARDIoGRAMplusC4D consortium data. This combines data from multiple large-scale genetic studies to identify risk loci for coronary artery disease and myocardial infarction [33, 35]. From the consortium we used two meta-analysis datasets to perform the lookup:

1. X-chromosome analysis dataset: Chromosome X-CAD [35]—meta-analysis of X-chromosomal variants for CAD including data from more than 43,000 CAD cases and 58,000 controls from 35 international study cohorts with random effects models. The X-chromosome dataset was used for *MTRNRL10* gene region lookup since it is located in the X-chromosome.
2. The second dataset was meta-analysis data of UK Biobank SOFT CAD GWAS (an interim release) with CARDIoGRAMplusC4D 1000 Genomes-based GWAS (i.e. dataset 4) and the Myocardial Infarction Genetics and CARDIoGRAM Exome (dataset 5) [33]. SOFT CAD phenotype was defined by the consortium as: fatal or nonfatal myocardial infarction, percutaneous transluminal coronary angioplasty or coronary artery bypass grafting, chronic ischemic heart disease, and angina. There were 10,801 cases and 137,914 controls.

eQTL analysis was carried out using the GTEx v7 data [34] from the GTEx Portal on 02/18/19 where lookup was conducted manually for all available tissues.

#### Description of analysis

For analysis we identified gene loci of 13 humanin-like nuclear isoform genes using the UCSC Genome Browser GRCh37/hg19 assembly [36]. We then performed a lookup for SNPs and eQTLs using the downloaded datasets.

1. SNP analysis:
  - The first analysis was done with the specific gene locus and the second analysis was done using the 50 kb flanking region in both directions.
  - As several variants were tested, multiple testing correction was used. The significance level for gene region analysis was calculated using Bonferroni correction; for the number of variants within the gene region it was 0.05/73 = 0.0007, and for the analysis with flanking regions it was 0.05/4909 = 0.00001.
2. eQTL analysis:
  A lookup was performed from the database for all available tissues. eQTL results were combined with SNP analysis results for reporting the hits present in both analyses.

### Results

We conducted a lookup for associations from 13 humanin-like nuclear isoform human gene regions located in different chromosomes using CARDIoGRAMplusC4D data [33, 35] (Table 1).

**Table 1:** Humanin-like nuclear isoform gene chromosomal position (GRCh37)

Gene	Chromosome	Position
MTRNR2L1	17	22022437–22023991
MTRNR2L2	5	79945819–79946854
MTRNR2L3	20	55933496–55934878
MTRNR2L4	16	3421053–3422283
MTRNR2L5	10	57358750–57360488
MTRNR2L6	7	142374131–142375525
MTRNR2L7	10	37890366–37891859
MTRNR2L8	11	10529434–10530723
MTRNR2L9	6	62284008–62284534
MTRNR2L10	X	55207824–55208944
MTRNR2L11	1	238107024–238108513
MTRNR2L12	3	96336030–96337067
MTRNR2L13	4	117220016–117221478

First, we looked for associations only within the regions of genes *MTRNR2L1*–*MTRNR2L13*, and second, we added 50 kb flanking regions in both directions to our lookup to extend the search for possible regulatory variants near these gene positions. The top variants for each region and from both lookups are shown in Table 2. There were no statistically significant associations in these gene regions after Bonferroni correction for the 73 variants found within the gene regions alone, nor for the 4909 variants found when these gene regions were searched with their added 50 kb flanking regions. The Bonferroni-corrected statistical significance limits were, respectively, 0.05/73 = 0.0007 and 0.05/4909 = 0.00001. The top variants within genes were rs78083998 from the *MTRNR2L13* region (meta-analysis P-value = 0.042) and rs11004929 from the *MTRNR2L5* region (P-value = 0.10). For lookup with flanking regions, the top variants were rs6151662 from the *MTRNR2L2* region (P-value = 0.0037) and rs76836360 from the *MTRNR2L8* region (P-value = 0.0044).

**Table 2:** Top variants from meta-analysis lookup in humanin-like nuclear isoform gene regions and with 50 kb flanking region in both directions

Gene	Top SNP (with flanking)	Alleles^a^	EAF	OR	P-value	Top SNP (no flanking)	Alleles	EAF	OR (95% CI)	P-value
				(95% CI)						
MTRNR2L1	rs56173058	G/A	0.98	1.08 (1.01–1.16)	0.034	rs79253571	C/T	0.98	1.04 (0.98–1.11)	0.17
MTRNR2L2	rs6151662	G/A	0.95	1.06 (1.02–1.11)	0.0037	rs150977859	T/C	0.053	1.02 (0.98–1.06)	0.3
MTRNR2L3	rs117764606	T/C	0.014	1.11 (1.03–1.20)	0.0074	rs113906620	T/C	0.025	1.04 (0.99–1.10)	0.14
MTRNR2L4	rs75856110	T/C	0.0026	1.34 (1.08–1.67)	0.0086	rs72776357	C/A	0.88	1.01 (0.98–1.03)	0.48
MTRNR2L5	rs72788864	G/A	0.05	1.05 (1.01–1.10)	0.016	rs11004929	C/T	0.37	1.01 (1.00–1.03)	0.1
MTRNR2L6	rs34587783	T/C	0.93	1.04 (1.01–1.08)	0.0087	rs58504986	A/G	0.56	1.01 (1.00–1.03)	0.14
MTRNR2L7	rs148910803	C/G	0.99	1.12 (1.00–1.24)	0.047	rs2208320	G/T	0.34	1.01 (0.99–1.02)	0.55
MTRNR2L8	rs76836360	T/C	0.00016	3.52 (1.48–8.37)	0.0044	rs7350542	G/A	0.19	1.02 (0.99–1.04)	0.15
MTRNR2L9	rs60678216	G/A	0.035	1.05 (1.01–1.10)	0.026	rs6915206	C/T	0.4	1 (0.99–1.02)	0.59
MTRNR2L10	rs5914251	C/A	0.14	1.02 (0.99–1.05)	0.15	rs10521478	A/G	0.57	1.01 (0.98–1.04)	0.62
MTRNR2L11	rs202137689	TG/-	0.03	1.06 (1.01–1.11)	0.029	rs12093187	C/T	0.029	1.04 (0.98–1.10)	0.25
MTRNR2L12	rs78077066	T/G	0.01	1.12 (1.02–1.23)	0.015	rs12106821	A/G	0.037	1.02 (0.95–1.10)	0.52
MTRNR2L13	rs10020248	C/T	0.21	1.03 (1.01–1.05)	0.013	rs78083998	G/C	0.031	1.05 (1.10–1.00)	0.042

^a^Effect allele/other allele

Additionally, we looked for eQTL variants of genes *MTRNR2L1*–*MTRNR2L13* from all available tissues using the GTEx database [34] and found 876 variants altogether. Out of these, 113 were present in CARDIoGRAMplusCD4D meta-analysis data. None of these exceeded the P-value threshold for Bonferroni multiple testing correction. The top marker was rs71476855 (P-value = 0.008) from the *MTRNR2L8* region. This variant showed eQTL association in thyroid tissue. Another marker, rs975494 from the *MTRNR2L6* region, is an eQTL in lung tissue (P-value = 0.047).

### Discussion

The lookup study for genetic association between coronary artery disease and humanin-like isoforms focused on genetic variants in different humanin-like peptide genes, both in the gene region and within the 50 kb flanking region we investigated, although none were statistically significant after multiple testing correction. The top variants in the flanking region analysis were in the *MTRNR2L2* and *MTRNR2L8* gene regions. These same genes were overexpressed in our previous study, and MTRNR2L8 has, already earlier, been shown to be expressed in heart tissue [11, 32]. Our results gave a possible eQTL SNP in the *MTRNR2L8* gene, which did not obtain statistical significance. It is possible that these isoform genes contribute to the omnigenic development of CAD, and factors influencing the expression of isoforms or the post-transcriptional and post-translational modification of isoforms may contribute to either the development or progression of CAD thereafter. Future studies should aim to answer these questions while specifying the role of humanin isoforms in cardiac cell metabolism, function and survival. Our study is the first that tries to answer whether single nucleotide variants in humanin-like nuclear isoform genes have an association with CAD.

The use of meta-analysis data from a multi-national consortium is one strength of this study. The number of participants is in the tens of thousands, which gives our study sufficient statistical power. Potential problem with phenotype association studies is that the definition of CAD is complicated due to differences in disease and symptom definitions, and coding systems. Furthermore, in this study, we only used the definition used by the original consortium, where CAD phenotype was defined as follows: fatal or nonfatal myocardial infarction, percutaneous transluminal coronary angioplasty or coronary artery bypass grafting, chronic ischemic heart disease and angina. It is possible that the results may differ when using a different phenotype definition.

Our study aimed to look for associations between DNA nucleotide variations in humanin-like nuclear isoform genes and established coronary heart disease. This lookup study showed no statistically significant associations between genetic variants in humanin nuclear isoform gene regions and coronary artery disease suggesting that these variants are not major contributors. They may influence disease development and progression in the omnigenic network.

## Limitations

- as the used consortium data did not contain mitochondrial DNA data we were not able to look for possible associations in the *MTRNR2* gene.
- We were only able to use the definition of CAD provided by the original consortium.
- Future studies should also address gender differences.

## Data Availability

the datasets analysed in this study are available for download on the CARDIoGRAMplusC4D consortium website (http://www.cardiogramplusc4d.org/) and on the GTEx Portal website (https://gtexportal.org/home/).

## Abbreviations

NCD: noncommunicable disease
CAD: coronary artery disease
IGFBP-3: insulin-like growth factor-binding protein-3
SNV: single nucleotide variant
SNP: single nucleotide polymorphism
eQTL: expression quantitative trait loci
